# Debate: Urban versus rural environments – which is better for mental health? Beyond the urban and rural dichotomy, a call to consider quality, typology and space in greenspace strategies for mental health

**DOI:** 10.1111/camh.12762

**Published:** 2025-03-10

**Authors:** Liza Griffin, Athina Petsou, Ruth Hynes, Gemma Moore

**Affiliations:** ^1^ The Bartlett Development Planning Unit University College London London UK; ^2^ Institute for Environmental Design and Engineering University College London London UK; ^3^ Independent Researcher London UK

**Keywords:** Greenspace, urban design and planning, mental health and wellbeing, urban, rural, typology

## Abstract

There is growing evidence about the positive impact of greenspaces on mental health and wellbeing. In their various forms, greenspaces such as parks, gardens, sports fields, and open spaces serve as crucial public amenities. This paper contributes to current debates on the mental health benefits of rural and urban environments by arguing for a nuanced and contextual understanding that goes beyond the urban–rural dichotomy, as access to these spaces has been shown to reduce stress and significantly improve mental wellbeing in both urban and rural settings. Through a critical examination of the literature, we focus on specific characteristics of greenspaces connected to mental health benefits. We then explore the ways in which specific urban environments and their qualities play crucial roles in mental health outcomes. The paper discusses the unique challenges and benefits of urban and rural greenspaces, emphasising the need for context‐specific strategies. We argue that urban planning and policy must prioritise the quality of greenspaces, particularly in urban environments, to recognise them as fundamental public health infrastructure and maximise their mental health benefits.

## Introduction

There is growing evidence about the positive impact of greenspaces on mental health and wellbeing. The term ‘greenspaces’ encompasses a wide variety of spaces covered by vegetation, such as street trees, gardens, landscaping around buildings, sports fields, flowerbeds, ponds, and parks. Many parks are ‘green’ but can also include open spaces like playgrounds and multi‐use game areas with little or no vegetation.

Despite their variety, these spaces are important public amenities, as access to parks and greenspaces has been shown to reduce stress and significantly improve overall mental wellbeing in both urban and rural environments. An evidence review from the World Health Organisation in 2021 found that the presence of greenspaces positively affected both short‐term and long‐term mental health outcomes (WHO, 2021). Studies have found that adults who live in green areas have lower rates of anxiety and depression, improved mental health, increased self‐reported life satisfaction and sense of worth and overall higher levels of happiness (Delgado‐Serrano, Melichová, Mac Fadden, & Cruz‐Piedrahita, 2024).

In this piece, we contribute to the debate on the mental health benefits of rural versus urban environments. We argue that by understanding how the specific characteristics of greenspaces are connected to mental health benefits—whether in urban or rural settings—and how greenspaces are used in daily life, we can develop a more nuanced understanding of how they are intertwined with mental health.

## Rural or urban?

While rural environments are generally greener, mental health needs are often greater in cities. City living is a risk factor for depression (40% increased risk), anxiety (20% increased risk), and schizophrenia (doubles the risk) (Peen, Schoevers, Beekman, & Dekker, 2010). Challenges to mental health in urban areas include loneliness, violence, high crime rates, homelessness, noise and other pollutants, traffic accidents, drug abuse, and insufficiency of mental health services. Rose and Fitzgerald (2015) ask:

Given that many experience urban stresses and strains – the hubbub, the noise, the competition, the density, the unnatural and frenzied atmosphere, the enforced proximity to strangers, the frequent combination of crowds and isolation – should we not be paying closer attention to the mental, and sometimes pathological, experience of city living itself?

The first answer to Rose and Fitzgerald's question might be: what kind of city living are we talking about? There are, of course, many different types of ‘urban’. As Stephens, Carrizo Gutierrez, and Ostadtaghizaddeh (2016) argue, the complex social, material, and economic matrix of each urban setting can have a unique effect on its inhabitants' lives and mental health. They argue that a city's size, its patterns of inequality and urbanisation, and people's access to quality environments together shape health outcomes.

The second answer might consider existing epidemiological evidence. While British studies show significant urban–rural mental health differences, this trend is not universal. For example, higher depression rates were found in rural Nigeria compared to urban areas (Amoran, Lawoyin, & Lasebikan, 2007). In South Africa, mental illness rates were highest in peri‐urban environments (Havenaar et al., 2008). Therefore, we should be mindful of generalising European or global North experiences. It is crucial to consider the context of mental health in rural and urban settings and the key qualities of different greenspaces.

## Quality or quantity?

A 2018 evidence review found that the total amount of greenspace was more strongly associated with mental health outcomes than the number of visits to greenspaces (Houlden, Weich, Porto de Albuquerque, Jarvis, & Rees, 2018). Another study found that the total area of greenspace in a neighbourhood was correlated with the rates of prescribed depression medication (Helbich, Klein, Roberts, Hagedoorn, & Groenewegen, 2018).

However, it seems that the quality of environments makes the most difference to people's health and wellbeing. It is now understood that communities with lower‐quality greenspaces are more likely to experience poorer health outcomes and higher rates of mental health problems, which in turn can increase the risk of unemployment (a significant social determinant of health), thereby creating a cycle which is difficult to break (Roe, Aspinall, & Thompson, 2016).

We, therefore, outline how the different qualities of greenspace and their use support good mental health. These include the importance of social connections, opportunities for physical activity, and direct engagement with nature. Understanding the degree to which these factors are present and can be afforded in different greenspaces, can help determine whether quality or quantity is more important in maximising their benefits.

## Social connections

Studies have shown that individuals living in greener areas report lower rates of loneliness and greater social cohesion, suggesting that greenspaces play a crucial role in fostering social relationships. There is little evidence on how these processes might be experienced differently in rural or urban environments, but there is ample discussion of their importance for mental health in cities. By providing accessible, shared spaces for recreation and interaction, greenspaces can facilitate community engagement, strengthen social networks, and reduce social isolation—important factors in promoting mental wellbeing, particularly in urban neighbourhoods. The driver of social connection appears to be the quality of greenspaces' social infrastructure.

## Physical activity

‘Green exercise’—physical activity in any natural environment—is known to reduce the risk of poor mental health, supporting psychological and physiological wellbeing (Barton & Rogerson, 2017). Urban and rural greenspaces both provide vital opportunities for movement, whether through walking, jogging, or group activities, making them essential public health resources. Numerous studies have further established strong links between physical activity in natural settings and improved mental health outcomes, reinforcing the importance of accessible, high‐quality greenspaces in both rural and urban areas (Houlden et al., 2018). The key determinant here seems to be the activities afforded, and the degree to which they are available to communities rather than their location.

## Connection to nature

Beyond social and physical benefits, direct exposure to nature itself has been linked to enhanced mental health. Research indicates that individuals with views of urban greenspaces from their homes or regular access to green pathways experience significantly lower levels of cortisol and stress (Honold et al., 2016). Engaging with nature—whether through passive experiences like observing greenery or active participation such as gardening—is associated with improved mood, reduced anxiety, and greater emotional resilience. Urban dwellers may have fewer opportunities than their rural counterparts to experience nature. However, research points to the manifold ways in which urban environments can also support meaningful nature connections and experiences of nature in cities can be just as meaningful for wellbeing.

## Conclusions

These social, physical, and biophilic mental health factors are mediated by different types of greenspaces. Multi‐use games areas and playgrounds may afford social connection and physical activity but not necessarily a connection to nature. Conversely, nature reserves may support biophilia and exercise but might be less effective for social interaction. Therefore, whether rural or urban greenspaces are better for mental health depends on the type and quality of the greenspace, the activities it supports, and the specific context of ‘the urban’ in question.

All this raises important questions about how to address the urban–rural divide in greenspace provision—should efforts focus on increasing the quantity and variety of greenspaces in cities, or does improving their quality hold the key to better mental health? Given the growing evidence base linking greenspaces to wellbeing and mental health outcomes through social connections, physical activity, and connections to nature, we believe that urban planning and policy must not only consider the quantity and variety of greenspaces available, but they must also prioritise maximising their quality—particularly in urban environments. Designing accessible, well‐maintained, and inclusive greenspaces can help augment the benefits, particularly for communities most at risk of poor mental health.

A shift towards health‐centred decision‐making is required, where greenspaces are recognised not simply as amenities but as fundamental public health and social infrastructure. To deliver these aspirations in cities, where they are arguably needed more acutely, cross‐sector collaboration between urban planners, mental health practitioners, and greenspace organisations is required. What is more, policies and plans must embed greenspaces in cities as a core component of mental health strategies in ways that consider their typology, quality and geography. This will necessitate community input so that greenspaces are more likely to meet the diverse needs of different urban populations.

## Funding information

There are no funders to report for this article.

## Ethics statement

No ethical approval was required for this debate article.

## Conflict of interest statement

The authors have declared that they have no competing or potential conflicts of interest.

## Data Availability

No ethical approval was required for this debate article.

## References

[camh12762-bib-0001] Amoran, O. , Lawoyin, T. , & Lasebikan, V. (2007). Prevalence of depression among adults in Oyo state, Nigeria: A comparative study of rural and urban communities. Australian Journal of Rural Health, 15, 211–215. 17542795 10.1111/j.1440-1584.2006.00794.x

[camh12762-bib-0002] Barton, J. , & Rogerson, M. (2017). The importance of greenspace for mental health. BJPsych International, 14, 79–81. 10.1192/s2056474000002051 PMC5663018 29093955

[camh12762-bib-0003] Delgado‐Serrano, M.M. , Melichová, K. , Mac Fadden, I. , & Cruz‐Piedrahita, C. (2024). Perception of green spaces' role in enhancing mental health and mental well‐being in small and medium‐sized cities. Land Use Policy, 139, 107087.

[camh12762-bib-0004] Havenaar, J.M. , Geerlings, M.I. , Vivian, L. , Collinson, M. , & Robertson, B. (2008). Common mental health problems in historically disadvantaged urban and rural communities in South Africa: Prevalence and risk factors. Social Psychiatry and Psychiatric Epidemiology, 43, 209–215. 18058040 10.1007/s00127-007-0294-9

[camh12762-bib-0005] Helbich, M. , Klein, N. , Roberts, H. , Hagedoorn, P. , & Groenewegen, P.P. (2018). More greenspace is related to less antidepressant prescription rates in the Netherlands: A Bayesian geoadditive quantile regression approach. arXiv, pp. 1–23. 10.1016/j.envres.2018.06.010 29936285

[camh12762-bib-0006] Honold, J. , Lakes, T. , Beyer, R. , & van der Meer, E. (2016). Restoration in urban spaces. Environment and Behavior, 48, 796–825.

[camh12762-bib-0007] Houlden, V. , Weich, S. , Porto de Albuquerque, J. , Jarvis, S. , & Rees, K. (2018). The relationship between greenspace and the mental wellbeing of adults: A systematic review. PLoS One, 13, e0203000. 30208073 10.1371/journal.pone.0203000 PMC6135392

[camh12762-bib-0008] Peen, J. , Schoevers, R.A. , Beekman, A.T. , & Dekker, J. (2010). The current status of urban‐rural differences in psychiatric disorders. Acta Psychiatrica Scandinavica, 121, 84–93. 19624573 10.1111/j.1600-0447.2009.01438.x

[camh12762-bib-0009] Roe, J. , Aspinall, P.A. , & Thompson, C.W. (2016). Understanding relationships between health, ethnicity, place and the role of urban greenspace in deprived urban communities. International Journal of Environmental Research and Public Health, 13, 1–21. 10.3390/ijerph13070681 PMC4962222 27399736

[camh12762-bib-0010] Rose, N. , & Fitzgerald, D. (2015). The metropolis and mental health: are big cities making us sick? *The Conversation*. Available from https://theconversation.com/the‐metropolis‐and‐mental‐health‐are‐big‐cities‐making‐us‐sick‐49264

[camh12762-bib-0011] Stephens, C. , Carrizo Gutierrez, A. , & Ostadtaghizaddeh, A. (2016). Revisiting the Virtuous City: Learning from the past to improve modern urban mental health. In N. Okkels , C. Blanner , P. Munk‐Jorgensen , P. Kristiansen , & M. Munk‐Jorgensen (Eds.), Mental health and illness in the city. Singapore: Springer.

[camh12762-bib-0012] World Health Organisation . (2021). Green and blue spaces and mental health: New evidence and perspectives for action. Last accessed 31 January 2025.

